# Inflammation and immunity in the pathogenesis of hypoxic pulmonary hypertension

**DOI:** 10.3389/fimmu.2023.1162556

**Published:** 2023-05-05

**Authors:** Yi Ye, Qiying Xu, Tana Wuren

**Affiliations:** ^1^ Research Center for High Altitude Medicine, Qinghai University, Xining, China; ^2^ High-Altitude Medicine Key Laboratory of the Ministry of Education, Xining, China; ^3^ Qinghai Provincial Key Laboratory for Application of High-Altitude Medicine, Xining, China; ^4^ Qinghai-Utah Key Laboratory of High-Altitude Medicine, Xining, China

**Keywords:** hypoxia, pulmonary hypertension, inflammation, immunity, metabolism, HIF

## Abstract

Hypoxic pulmonary hypertension (HPH) is a complicated vascular disorder characterized by diverse mechanisms that lead to elevated blood pressure in pulmonary circulation. Recent evidence indicates that HPH is not simply a pathological syndrome but is instead a complex lesion of cellular metabolism, inflammation, and proliferation driven by the reprogramming of gene expression patterns. One of the key mechanisms underlying HPH is hypoxia, which drives immune/inflammation to mediate complex vascular homeostasis that collaboratively controls vascular remodeling in the lungs. This is caused by the prolonged infiltration of immune cells and an increase in several pro-inflammatory factors, which ultimately leads to immune dysregulation. Hypoxia has been associated with metabolic reprogramming, immunological dysregulation, and adverse pulmonary vascular remodeling in preclinical studies. Many animal models have been developed to mimic HPH; however, many of them do not accurately represent the human disease state and may not be suitable for testing new therapeutic strategies. The scientific understanding of HPH is rapidly evolving, and recent efforts have focused on understanding the complex interplay among hypoxia, inflammation, and cellular metabolism in the development of this disease. Through continued research and the development of more sophisticated animal models, it is hoped that we will be able to gain a deeper understanding of the underlying mechanisms of HPH and implement more effective therapies for this debilitating disease.

## 1 Introduction

Hypoxic pulmonary hypertension (HPH) (1), also known as WHO group III pulmonary hypertension (PH), can be caused by exposure to high altitudes or chronic lung diseases that lead to systemic hypoxia, which is a heterogeneous group of diseases that leads to a continuous increase in pulmonary artery pressure (PAP) and significant progression of pulmonary vascular remodeling in the presence of alveolar hypoxia (2).

With a combination of targeted drugs (3) such as phosphodiesterase-5 inhibitors, endothelin receptor antagonists, prostacyclin analogs, and prostacyclin receptor agonists, patients with pulmonary arterial hypertension (PAH, i.e., WHO group I PH) have shown a significant improvement in activity tolerance and disease prognosis. However, clinical trials of these drugs have not shown any beneficial effects in patients with HPH (4, 5). Therefore, clarifying the underlying pathogenic mechanisms in this group of patients will help to improve targeted therapies.

To date, researchers have begun to study the role of perivascular inflammation in the onset of HPH, and there is mounting evidence that hypoxia, immune-cell inflammation, and metabolic imbalance in vascular cells play a role in its progression and development. High altitude or hypoxic exposure can also lead to PH as they affect immune homeostasis and regulatory activity.

This review presents evidence of immune-inflammatory dysregulation occurring in pulmonary arteries during chronic hypoxia exposure, including hypoxia-induced hyperproliferation/hypertrophy of resident vascular cells, infiltration of immune cells, and expression of pro-inflammatory factors. It also emphasizes that metabolic reprogramming under the hypoxic microenvironment triggers functional changes in the inflammatory response and immune cell activity, suggesting the importance of hypoxia in the initiation and progression of pulmonary vascular lesions and inflammation.

**Figure 1:**
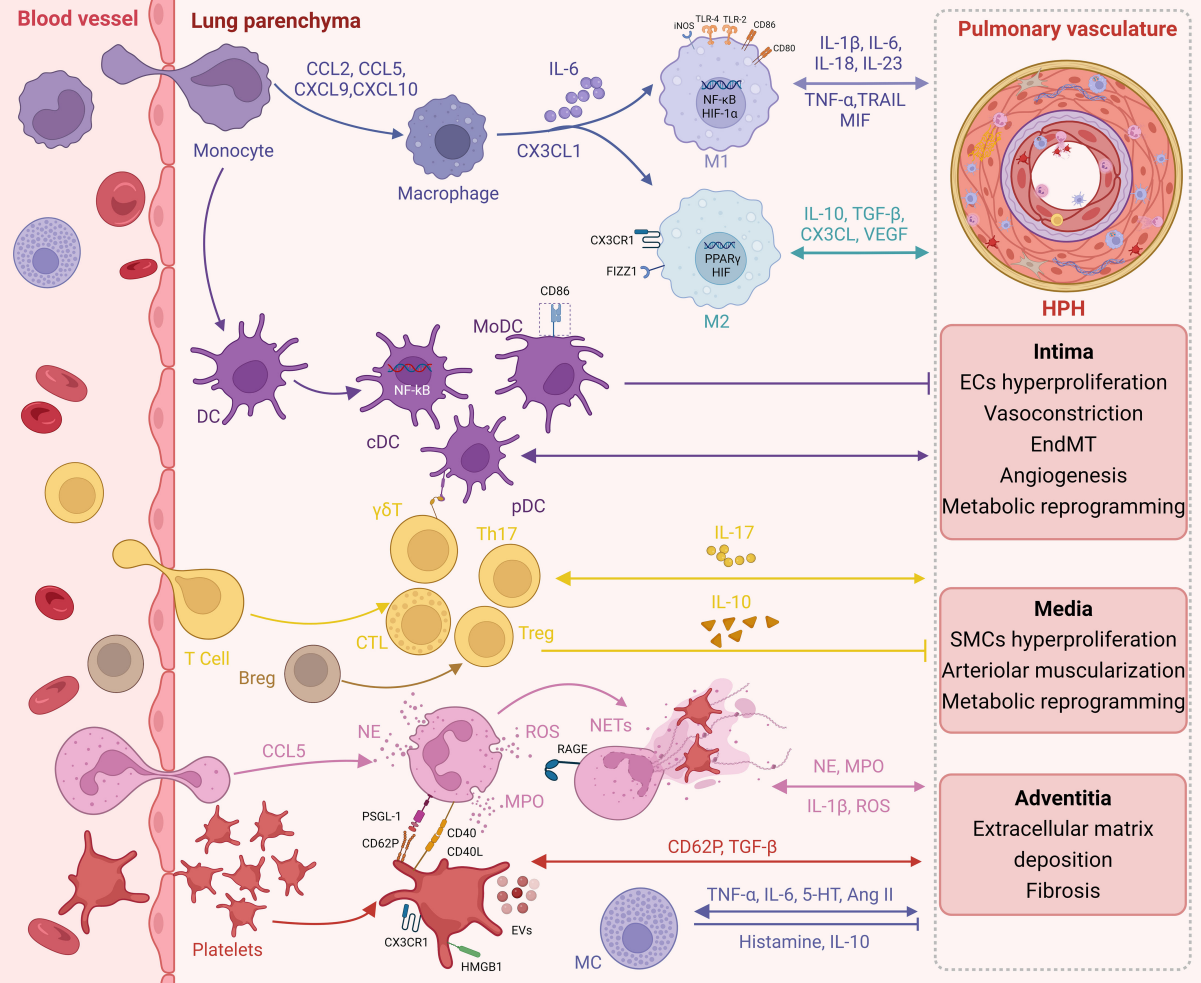
Immunoinflammation in HPH. Infiltration of circulating immune cells and expression of pro-inflammatory factors during chronic hypoxic exposure promote immune inflammatory dysregulation in the pulmonary arteries. In turn, long-term resident vascular cell dysfunction further promotes an immune inflammatory response.

In this review, we provide evidence of immune-inflammatory disruption in pulmonary arteries upon chronic exposure to hypoxia, characterized by hyperproliferation/hypertrophy of resident vascular cells, in addition to the recruitment of immune cells and upregulation of pro-inflammatory agents. These results indicate the significance of hypoxia in the development and progression of pulmonary vascular lesions and inflammation (**Figure 1**).

## 2 Evidence for inflammatory infiltration in HPH

The presence of inflammation in HPH has been shown in histological results (6); inflammatory infiltrates consisting of macrophages, mast cells (MCs), neutrophils, platelets, T- and B-lymphocytes, and dendritic cells have been found around pulmonary vascular lesions. These studies indicate that perivascular inflammation could significantly correlate with intimal and medial fractional thickness, leading to increased PAP (7) and contributing to the structural remodeling of pulmonary circulation.

Oxygen deprivation in the lung tissue is the basis of HPH vasculopathy. Hypoxia inducible factor (HIF) is a major transcription factor involved in oxygen-sensing homeostasis, which contributes to the pathogenesis of HPH (8). HIF is responsible for the activation of signaling pathways in pulmonary arterial endothelial cells (PAECs) (9) and pulmonary artery smooth muscle cells (PASMCs), which have a direct influence on cell proliferation (10); it also orchestrates inflammatory dysregulation in response to hypoxia levels, which in turn induces profound changes in endothelial and smooth muscle cell phenotypes.

HPH is caused by chronic hypoxia, which can be primarily or secondarily caused by a lack of oxygen in the cellular microenvironment owing to an inflammatory response. HIF promotes aggregation of inflammatory cells and release of inflammatory mediators. Immune cells exposed to a hypoxic environment leak from the oxygen-enriched blood to the site of inflammation, which activates and stabilizes HIF. For example, when macrophages and neutrophils infiltrate HPH, HIF-1α is known to increase macrophage proliferation, chemotaxis, and infiltration, and drive the release of cytokines (11). Under hypoxia, the inhibition of mitochondrial oxidative phosphorylation and altered electron transport chain causes an increase in macrophage reactive oxygen species (ROS) production, which activates and stabilizes HIF-1α (12). HIF-1α also increases neutrophil extracellular traps (NETs) formation (13) and neutrophil survival by inhibiting apoptosis and triggering the nuclear factor NF-κB signaling pathway (14). Activated neutrophils stimulate the expression of HIF-1α and HIF-2α (15).

In contrast, activation of HIF-centered hypoxic signaling pathways can initiate immune inflammatory responses. The key regulators of transcriptional responses in hypoxia and inflammation are HIF and NF-κB (16), respectively, both of which promote positive feedback loops in pulmonary vascular remodeling. The p65 and p50 subunits of NF-κB efficiently bind to the HIF-1α promoter thereby enhancing HIF-1α (17) expression. Jin et al. (18) found that, in response to hypoxia, endothelial cells can efficiently secrete TNF-α, which in turn leads to the activation of HIF through the NF-κB pathway, ultimately boosting vascular endothelial growth factor (VEGF) production. After a reduction in NF-κB pathway participant expression, the TNF-α/NF-κB/HIF signaling cascade was inhibited, causing HIF-dependent inactivation of downstream events and ultimately hypoxia-induced inhibition of angiogenesis. HIF-1 increases its expression by binding to the promoter region of CD146. Therefore, an increase in CD146 levels stimulates the HIF-1 transcriptional pathway through NF-B activation (19), leading to hypoxia-induced cell proliferation and anti-apoptosis. Thus, elevated CD146 in PASMC correlates with the severity of PH. CD146-mediated cross-regulation of HIF-1α with NF-κB promotes the synthetic phenotype of PASMCs. Chronic hypoxia triggers pulmonary vasculopathy and NF-κB expression, and NF-κB inhibition reverses hypoxia-induced pulmonary vascular remodeling and right ventricular hypertrophy (20).

There is a strong link between hypoxia and the HIF immune inflammatory response; therefore, HIF-mediated immune cell dysregulation and HIF/NF-κB crosstalk are jointly involved in the vascular pathology of HPH. The formation of HPH begins with hypoxia-triggered pulmonary vascular inflammatory dysfunction, which is a cause rather than a consequence of the development of PH. Along with increased perivascular aggregation and intravascular infiltration of immune cells, hypoxia-induced vasoconstriction and hyperproliferation are exacerbated by elevated circulating cytokines and chemokines (21), highlighting the importance of their involvement in vasculopathy in the pathogenesis of HPH.

## 3 Immune cells and inflammatory factors in HPH

Recent research indicates that inflammatory and vascular cells interact in vascular lesions associated with HPH. Inflammatory cells not only participate in the pathological process of HPH through direct contact with pulmonary vascular cells but also release cytokines and chemokines to contribute to the exaggerated contractility and proliferation of vascular cells.

### 3.1 Macrophages

The most prominent type of inflammatory cells in the alveolar and pulmonary vessel walls is macrophages (22), which play a role in the vascular remodeling process associated with chronic hypoxia. Given the tendency of macrophages to be recruited to areas of poor vascularity with low oxygen tension (23), the primary function of macrophages in the regulation of HPH (24) deserves further investigation.

Many studies have revealed early and persistent accumulation of macrophages in perivascular lesions (24, 25). The significance of macrophages in PAP (26) and pulmonary vascular remodeling has been validated using targeted interventions. Macrophage depletion normalized mean pulmonary artery pressure (mPAP) and restored right ventricular systolic pressure (RVSP) in bone morphogenetic protein type-II receptor (BMPR-II) knockout mouse models subjected to chronic hypoxia (27).

Macrophages can adapt to their immune microenvironment by undergoing polarized activation, which may be roughly classified into two types: conventionally activated macrophages (M1) and alternatively activated macrophages (M2) (28). By encouraging the release of pro-inflammatory molecules, M1 enhances inflammation, whereas M2 stimulates proliferation and angiogenesis (29). HPH patients and animal models activated both M1 and M2, but M2 predominated (25). Inducing inflammation and pulmonary vascular remodeling, M2 accelerates PAH development (30). Macrophage polarization was affected by sex disparity, and macrophage-depleted HPH mice confirmed that HPH progression was associated with M2 predominance in the lungs of male mice (31), and that this M1/M2 ratio imbalance was mediated by interleukin 6 (IL-6) (32).

It is essential for the development of HPH that macrophages are recruited early and activated in hypoxic lungs, leading to the destruction of blood vessels and their remodeling (21). In hypoxia, chemokines such as fractalkine/CX3CL1, which cause the adhesion of CX3CR1 expressing immune cells (particularly macrophages) to PAECs, lead to dysfunction of PAECs and initiation of a perivascular inflammatory response (33). By influencing monocyte recruitment, macrophage polarization, and PASMCs proliferation in the lungs, CX3CR1 plays a significant role in the development of HPH (24). This was demonstrated by the progression of PH being slowed in mice lacking the CX3CR1 gene (25).

Under hypoxic exposure, activated PAECs trigger the production of IL-6 (34), Chemokine (C-C motif) ligand 2 (CCL2) (35), matrix metalloproteinase (MMP)-9 (36), platelet-derived growth factor-B (37), and leukotriene B4 (LTB4) (38), and the accumulation of these known inflammatory mediators may produce a pro-inflammatory microenvironment that is detrimental to the integrity of the pulmonary vascular bed, thereby further worsening vascular remodeling.

The aggregation of macrophages surrounding pulmonary artery vessels is a hallmark of HPH. Macrophage-mediated lung inflammation is a prime cause of lung remodeling and, by extension, of right ventricular remodeling. New therapeutic options for HPH require a better understanding of the role of macrophages in immunological responses to the disease.

### 3.2 Mast cells

Accumulation of mast cells (MCs) around blood vessels was found to be positively associated with right ventricular hypertrophy in chronically hypoxic pigs, rats, and sheep (39). Further research has found that hypoxia induces an increase in MCs in the lungs of rats and humans, leading to pulmonary vascular remodeling and HPH (40, 41).

Hypoxia causes recruitment, activation, and degranulation of MCs, releasing compounds that promote PH and pulmonary vascular remodeling (42), such as tumor necrosis factor alpha (TNF-α), IL-6 (43), trypsin, chymotrypsin, 5-HT, and Ang II. Targeting MCs in PH may reduce PAP and pulmonary vascular remodeling (44). However, MCs can also reduce inflammation and suppress the immune system (45). Recovery from chronic hypoxia is associated with the reversal of pulmonary vascular remodeling, which is linked to an increase in collagenase in pulmonary MCs and collagenolytic and elastolytic activities in lung tissue (46). More research is needed to understand the dual involvement of MCs in increasing inflammation and dampening immune responses in HPH.

### 3.3 Neutrophils

Neutrophils have long been thought to be terminally differentiated cells, but little attention has been paid to their function in the pathophysiology of HPH. As first-line effectors of host defense, they need the capacity to detect low oxygen tension in the environment and adapt by altering a number of crucial processes (47). Cells may adapt by increasing their degranulation in response to low oxygen levels. A variety of regulatory inputs may alter neutrophils to alter HIF activity at hypoxic inflammatory sites (48). This suggests that neutrophils play a larger role in the pathophysiology of HPH than previously thought.

It has been shown that PH patients and animal models, have significantly elevated levels of neutrophils (49), associated with clinical indices of disease progression such as increased pulmonary vascular resistance (7) and decreased 6-minute walk distance (6 MWD) (50). Recent research has pointed to the neutrophil-to-lymphocyte ratio (NLR) as a possible inflammatory indicator that may be used as a marker of systemic inflammation in a variety of conditions (51), including PH. NLR and pulmonary vascular resistance are strongly correlated in individuals with PAH (52) or sarcoidosis-associated PH (53), also known as WHO Group V. Similarly, NLR predicts preoperative pulmonary vascular resistance and surgical mortality in patients with chronic thromboembolic PH (54), also known as WHO Group IV.

In both pre- and clinical studies of PH, more neutrophils were found in blood and lung tissues. However, the increase in neutrophils was different in each PH group, with the most noticeable change occurring in HPH (55). The activation of recruited neutrophils leads to further recruitment of cellular mediators, secretion of cytokines, and release of neutrophil elastase (NE) (56) and myeloperoxidase (MPO) (57).

Neutrophils infiltrating the pulmonary vasculature of mice with chronic hypoxia-induced PH were able to secrete IL-1β (58), and knockdown of IL-1β receptors in mice or use of IL-1β receptor inhibitors attenuated the persistent elevation of PAP in an animal model of PH, resulting in a significant reduction in RVSP (59). In PASMCs, IL-1β can inhibit the conversion of ATP to cAMP by downregulating the activity of adenylate cyclase (60). IL-1β modulates the growth of PASMCs through the IL-1R1/MyD88 pathway (61). IL-1β released by neutrophils may directly control hypoxia-stimulated vasoconstriction and remodeling in pulmonary arteries. In the pathological condition of HPH, neutralization of IL-1β, inhibition of IL-1β signaling, or suppression of upstream pathways regulating IL-1β secretion may be effective in attenuating disease progression.

Nickel et al (56) observed increased NE activity in the pulmonary vasculature of a rat model of SU5416/hypoxia (Su/Hx)-induced PH and that inhibition of NE activity by application of elafin (an endogenous NE inhibitor) improved endothelial function, reduced small pulmonary artery remodeling, and decreased PAP, emphasizing that inhibition of NE activity offers new insights for the therapy of HPH. By reducing MPO in rats with Su/Hx-induced PH, MPO exacerbates pulmonary vasoconstriction and pulmonary vascular remodeling by activating the RhoA/Rho kinase pathway (57), suggesting that MPO may be a precipitating factor in HPH.

Recent studies have shown a functional relationship between NETs and inflammatory angiogenesis *in vitro* and *in vivo* and a correlation between their formation and pulmonary vascular remodeling (62). NETs may promote pulmonary vascular remodeling *via* multiple mechanisms, and their mediated imbalance in pulmonary vascular homeostasis may have implications for a variety of different types of PH diseases. In hypoxia, dysfunction of pulmonary vascular wall cells leads to increased production of ROS (63), which NETs are dependent on (62).

Elevated cytokine levels in HPH enhance neutrophil population and function. Neutrophils, after being recruited to lung tissue during HPH, are not only involved in perivascular inflammation (64) but also mediate pulmonary vascular remodeling through the degranulation and release of cytokines and proteases, as well as the formation of NETs (65).

### 3.4 Platelets

As the second most abundant cell population in peripheral blood, platelets are involved not only in the hemostatic process but also in the repair of endothelial cells, promotion of cell growth, and the development of inflammatory diseases (66). The immune function of platelets depends on the precise balance between pathogenic and hemostatic effects and their thrombogenic actions. Several studies have shown that platelets are involved in the onset and development of HPH and are associated with its prognosis of HPH (67–70). Platelet distribution width (PDW), a measure of platelet activation, has been proposed as a risk predictor of PAH (71). In patients with severe HPH, in-hospital PDW was independently related to all-cause death, suggesting that PDW may be a predictive indicator for platelet activation and severity (72). The platelet-to-lymphocyte ratio (PLR) is a novel inflammatory marker that can be used to predict inflammation and mortality in various diseases. As a combination of platelets and lymphocytes, the PLR has been shown to correlate with the severity and prognosis of HPH (73).

As markers of platelet surface activation, P-selectin (also known as CD62p) and glycoprotein (GP)IIb/IIIa are highly expressed in the Su/Hx mouse model (74), clearly demonstrates the activation of platelets in HPH. P-selectin can bind to NEDD9 to facilitate hypoxia-sensitive pulmonary vascular remodeling through platelet-endothelial cell adhesion (75), a new mediator of HIF-1 dependent PAECs (76). Additionally, activated platelet supernatant promotes PASMCs metabolic reprogramming *via* the release of transforming growth factor-β (TGF-β) (74).

Platelets and neutrophils interact through the binding of multiple ligands/receptors, involvement of chemokines, and extracellular vesicle-mediated effects to promote mutual activation (77) at the tissue and systemic levels. HMGB1/HMGB1 receptors (69, 70), TLR4, and RAGE (78) levels were significantly higher in HPH, implicating an underlying platelet-neutrophil interaction in the pathogenesis of HPH. The binding of the chemokine fractalkine to the platelet-specific receptor CX3CR1 stimulates neutrophil activation and causes perivascular inflammation, leading to abnormal constriction of small pulmonary arteries, vascular remodeling, and increased PAP. The neutrophil chemokines CXCL4 and CCL5 were significantly reduced in PH mice with reduced platelet specificity (79). Among neutrophil–platelet interactions, the modulation of NETs is of great interest (80). Although few studies have been conducted on platelet-neutrophil interactions in PH disease progression, the current evidence highlights the dual-mediated inflammatory response and prothrombotic state as an underlying part of HPH disease progression.

As a result, platelets contribute to the formation and progression of HPH through aggregation, activation, and promotion of thrombosis, leading to increased pulmonary vascular resistance through secretion of various pro-vasoconstriction factors, growth factors, inflammatory mediators, and production of EVs, causing pulmonary artery constriction and proliferation of vascular component cells through recruitment of various inflammatory cells (especially neutrophils) to promote local injury. Our previous study confirmed that in high-altitude polycythemia, a type of altitude sickness, hypoxia leads to an increase in platelet activation and procoagulant factors (81), again supporting platelet involvement in HPH.

### 3.5 T Cells, B Cells, and DCs

Based on their function and surface markers, T lymphocytes can be classified as helper T cells (Th cells), regulatory T cells (Tregs), and cytotoxic T cells (CTLs). Th17 cells lead to an increase in perivascular T cells and are involved in the development of HPH due to their anti-apoptotic effects (82) and their ability to enhance the proliferation and migration of PASMCs (83). Th17 cell development was also observed. The Th1 subset produces inflammatory mediators and colocalizes with Th17 in the pulmonary vascular region (84), likely contributing to HPH progression through mutual recruitment. Tregs can protect mice from HPH by inhibiting the inflammatory response, promoting the release of anti-inflammatory factors such as IL-10 (85) and reversing the effect of hypoxia on the cell cycle (86). Hypoxia is known to mediate pulmonary vascular remodeling *via* activation of the Akt/Erk signaling pathway (87). Tregs also downregulate the phosphorylation of Akt and ERK (86), thereby improving hemodynamic parameters and vascular remodeling in HPH. Tregs and macrophages potentially interact in HPH; activated macrophages can increase LTB4 secretion in the absence of Tregs, leading to changes in vascular cell phenotype (88). Tregs suppress adaptive immune responses; there is an imbalance in the Th17/Treg ratio in patients with PH associated with chronic obstructive pulmonary disease (COPD-PH), and the Th17/Treg balance axis trends towards the Th17 cell lineage (89). The pathophysiology of PH is associated with the dysregulation of circulating CTLs and their granulysin, but its function is not well understood (90, 91). Therefore, T lymphocytes play a significant role in the progression of HPH through their interactions with different signaling pathways and their ability to promote or inhibit inflammation; however, further research is needed to fully understand the role of T cells as therapeutic targets.

The role of B cells in HPH was previously understood to merely regulate antibody production (92). However, recent studies have suggested that a subset called regulatory B cells (Bregs) has additional functions including immunosuppression (93). Bregs have been shown to prevent HPH by regulating the immune balance of Tfh/Tfr and inhibiting the proliferation of PASMCs under hypoxic conditions (94).

Dendritic cells (DCs) play a role in regulating the inflammatory response and have been found in lung samples from IPAH patients and their corresponding animal models (95). Subgroups of DCs have varying effects on the pulmonary vascular immune microenvironment. For example, activated conventional dendritic cells (cDCs) promote inflammation *via* the NF-κB pathway and development of PH (96). PAH patients show reduced expression of the co-stimulatory factor CD86 on monocyte-derived dendritic cells (MoDCs), leading to impaired function in the PAH-initiated immune or immune tolerance state (97). Plasmacytoid dendritic cells (pDCs) are increased in the lungs of PAH patients (98) and likely act in the tissue immune microenvironment of PH through crosstalk with γδT cells (99).

## 4 Cytokines, chemokines, and HPH

In hypoxia, dysfunction of pulmonary vascular wall cells and activation of inflammatory cells leads to an increased release of pro-inflammatory mediators. Hypoxia may be a link between cytokines and chemokines and the vascular pathology of HPH. **Table 1** provides a summary of the evidence for the involvement of various cytokines/chemokines in the pathogenesis of HPH, including both preclinical (animal models of disease) and clinical findings.

**Table 1:** Cytokines and chemokines associated with pulmonary vascular inflammation.

Cytokines/Chemokines	Reference	Cellular Source	Targeted Cells	Function in HPH
IL-1α	(100)	PAECs PASMCs	PASMCs	Enhancement of IL-1β expression Promotion of PASMC proliferation
IL-1β	(101–104)	Monocytes/macrophages PAECs PASMCs Fibroblasts Lymphocytes	Macrophages PASMCs	Recruitment of macrophages Promotion of PASMC proliferation and anti-apoptosis Induction of pyroptosis
IL-6	(105–113)	Monocytes/macrophages PAECs PASMCs Fibroblasts T- and B-cells	Lymphocytes Th17 Macrophages PAECS PASMCs	Promotion of PAEC proliferation and of PASMC apoptosis resistance Recruitment of lymphocytes Differentiation of Th17 Polarization of macrophages Downregulation of BMPR-II
IL-10	(114, 115)	TH1- and TH2-cells CTLs B-cells MCs Macrophages	Macrophages PASMCs	Reduction of macrophage infiltration Decrease expression of TGF-β and IL-6 Inhibition of excessive PASMC proliferation
IL-17	(83, 116, 117)	Th17	Th17& Tregs PAECs	Imbalance of the Th17/Treg ratio Promotion of PAEC proliferation Angiogenesis
IL-18	(101, 104)	Macrophages DCs	Macrophages PASMCs	Functions synergistically with IL-1β
IL-33	(118)	PAECs Fibroblasts	PAECs	Enhances proliferation, adhesion and spontaneous angiogenesis of PAECs
TNF-α	(119–121)	Many cells including monocytes/macrophages, PAECs	PASMCs	Promotion of GM-CSF secretion Promotion of the proliferation of PAECs and PASMCs Repression of BMPR-II in PAECs and PASMCs
TRAIL	(122–124)	Monocytes/macrophages	PASMCs	Promotion of PASMC proliferation Activation of NF-κB signaling
TGF-β	(125–131)	PASMCs, fibroblasts, monocytes, some T cells, B-cells,	PAECs PASMCs	Proliferation of PASMCs Deposition of extracellular matrix EndMT Dysregulation of TGF-β and BMP signaling pathways
MIF	(132–135)	Macrophages, lymphocytes, ECs, SMCs, and epithelial cells	PAECs PASMCs Fibroblasts	Hyperproliferation of PAECs, PASMCs and, fibroblasts Vasoconstriction
HIMF	(136–140)	Alveolar epithelium Fibroblasts	PASMCs PAECs Macrophages	Induction of inflammatory mediator production Proliferation of PASMCs and PAECs Mediates HMGB1/RAGE signaling Activation of HIF and NF-κB signaling Warburg effect
HMGB1	(69, 141, 142)	Necrotic PAECs, PASMCs and innate immune cells	PASMCs	Enhancement of the expression of IL-6 Downregulation of BMPR-II Promotion of proliferation and migration of PASMCs
CCL2/MCP-1	(25, 143–145)	PAECs PASMCs	monocytes/macrophages PASMCs Fibroblasts	Chemotactic monocytes/macrophages Activation of PASMCs and fibroblasts
CCL5/RANTES	(35, 146, 147)	T cells, PAECs, PASMCs, macrophages, fibroblasts, epithelial cells	monocyte/macrophage PASMCs PAECs	Pro-inflammatory Enhancement of monocyte/macrophage adhesion to PAECs and macrophage infiltration Induction of pulmonary angiogenesis and occlusion Promotion proliferation and migration of PASMCs
CXCL9	(148, 149)	Monocytes	PAECs	Induction of PAEC apoptosis
CXCL10	(148, 149)	Monocytes	PAECs	Induction of PAEC apoptosis
CXCL12	(150–153)	Bone marrow stromal cells, fibroblasts	PASMCs	Promotion of PASMC proliferation and interference with cell cycle progression
CX3CL1/Fractalkine	(25, 33, 154)	PAECs, DCs, macrophages, T cells, SMCs	Monocytes/macrophages γδ T cells DCs Pericytes PASMCs	Stimulation of leukocyte capture, chemotaxis and transport Monocyte recruitment Macrophage polarization Promotion of proliferation in pericytes and PASMCs

### 4.1 Cytokines

The IL-1 family of cytokines plays a crucial role in the inflammatory response, particularly in promoting excessive proliferation of PASMCs (155). In animal models of HPH, higher levels of IL-1α and IL-1R1 (specific receptors of IL-1a, IL-1b, and IL-1Ra) have been detected in lung tissues, lesions in pulmonary vessels, and serum (100). Treatment with IL-1R1 knockout or anakinra, an IL-1R1 antagonist, protected mice from HPH.

IL-1β and IL-18 (101) have been shown to regulate the recruitment of pulmonary perivascular macrophages, resist hypoxia-induced pyroptosis (102), and synergistically activate the caspase-1/STAT3 pathway, promoting macrophage-based inflammatory cell infiltration (103). Furthermore, serum levels of IL-1β and IL-18 (104) are reliable predictors of pulmonary vascular remodeling and HPH risk. IL-33 has also been found to enhance HPAEC proliferation, adhesion, and spontaneous angiogenesis in an HIF-1α-dependent manner, resulting in vascular remodeling and HPH (118).

IL-6 is one of the most significant players in the pathophysiology of HPH and is highly expressed in plasma (105), lung tissue, and the vascular media of PASMC from HPH patients and animal models (106). Lung-specific IL-6 overexpressing mice developed PH spontaneously under chronic hypoxic conditions and show distal small arterial muscularization. Animals with knocked out IL-6 receptors (107), as well as mice lacking IL-21 receptors, are resistant to HPH (34). After exposure to hypoxia, there was a boost in IL-6 mRNA levels in the lungs, and delivery of recombinant IL-6 protein induced vascular remodeling and enhanced the response of HPH (108).

IL-6 signals *via* glycoprotein 130 (gp130) and membrane-bound IL-6 receptor (mIL-6R) or soluble IL-6 receptor (sIL-6R), referred to as classic or trans-signaling, respectively. This induces anti-inflammatory and pro-inflammatory responses, thereby initiating its pleiotropic effects (109). Recent studies suggest that IL-6 trans-signaling contributes to HPH by (1) IL-6 induced excessive proliferation of PAECs and proliferative apoptotic resistant phenotype of PASMCs in the distal pulmonary vascular wall in HPH through upregulation of vascular endothelial growth factor receptor 2 (VEGFR2), MMP-9 expression, and lymphocyte recruitment (110); (2) BMPR-II was found to be significantly downregulated in rodents exposed to hypoxia (111); IL-6 is responsible for BMPR-II downregulation through the transcriptional activator STAT3-microRNA cluster 17/92 pathway (112); and (3) IL-6 potentiates hypoxia-induced PASMC migration induced by TH17 cells and promotes the infiltration of M2 macrophages around lesional pulmonary vessels (113).

This suggests a role for IL-6 in the regulation of pulmonary vascular inflammation and myelination in HPH through immune and inflammatory responses mediated by pro-proliferative anti-apoptotic mechanisms.

IL-10 is linked to the pathogenesis and progression of COPD-PH, and a decrease in IL-10 levels is a risk factor for the development of COPD-PH (114). Previous studies have shown that exogenous IL-10 prevents proliferative vasculopathy, PASMCs proliferation, and chemokine expression *in vivo* by inhibiting inflammatory cell infiltration to prevent Monocrotaline-induced PH (115). The pathogenic mechanism of IL-10 in HPH requires further investigation. Th17 induces an altered vascular cell phenotype in HPH by a mechanism of inflammatory cell infiltration and vascular cell proliferation (82), and the Th17-produced pro-inflammatory cytokine IL-17 expression is upregulated in both bronchodilator/COPD-PH patients and HPH model mice (116). Hypoxia upregulates IL-17 expression (117), which mediates pulmonary vascular remodeling by affecting cell proliferation, angiogenesis, and adhesion function of PAECs in HPH (83).

TNF-α, a pro-inflammatory cytokine, was significantly elevated in the medial layer of the pulmonary artery in patients with COPD-PH (156) and in the Su/Hx rat model (119), confirming the involvement of TNF-α as an inflammatory indicator of HPH pathology. TNF-α drives PAECs and PASMCs proliferation in HPH by inhibiting pyruvate dehydrogenase (PDH) activity (120), suppressing BMPR-II, and altering NOTCH signaling. Rats treated with the TNF-α antagonist, recombinant TNF-α receptor II, and IgG Fc fusion protein (rhTNFRFc) (121) showed some improvement in pulmonary hemodynamics and lung inflammation, which may provide alternative therapeutic targets for HPH treatment.

TNF-related apoptosis-inducing ligand, also known as TRAIL, is a potent stimulator of pulmonary vascular remodeling in human cells and rodent models. In rodent models of HPH, TRAIL depletion or blockade is associated with reduced pulmonary artery remodeling and reduced PASMC proliferation (122). TRAIL knockdown has a similar protective effect in HPH mouse model (123). In addition, TRAIL/TRAIL receptors transduce pro-inflammatory signals through the activation of NF-κB signaling (124), thus participating in the inflammatory response of HPH.

TGF-β is a highly polytropic cytokine that regulates inflammatory processes, particularly in the cardiovascular system. Activation of TGF-β is both necessary and sufficient for the development of PH in chronically hypoxia-exposed mice (125). According to research, increased TGF-β signaling (126) and decreased BMP signaling (127) are involved in the occurrence and development of HPH by promoting PASMCs proliferation (128), extracellular matrix deposition (129) and EndMT (130). These processes were dominated by PASMCs and PAECs. TGF-β is a crucial regulator of pulmonary vascular remodeling and inflammation in addition to cardiac hypertrophy and fibrosis. Selective TGF-β ligand blockade (131) in multiple experimental HPH models improves hemodynamics and remodeling as well as RVSP, right ventricular function, and survival.

Macrophage migration inhibitory factor (MIF) is a multifunctional cytokine that regulates inflammation and immune responses. MIF expression was increased in PASMCs and PAECs of HPH rats (132). MIF enhances hypoxia-induced pulmonary vasoconstriction (133) in addition to participating in hypoxia-induced pulmonary vascular remodeling by promoting the proliferation of ECs (134), SMCs, and fibroblasts (135).

Hypoxia-induced mitogenic factor (HIMF/RELM/FIZZ1) expression was positively correlated with elevated PAP in the remodeled pulmonary vascular system (136). HIMF stimulates pulmonary vascular cells to produce high levels of pro-inflammatory mediators (137), such as HIF-1, IL-6, MCP-1, and ROS, which enhance perivascular immune cell recruitment. Among them, HIF-1 is a major transcription factor involved in HIMF-induced pulmonary vascular remodeling and the development of PH. HIMF has been shown to (1) directly stimulate proliferation of SMCs in HPH through activation of the MAPK signaling pathway (138); (2) stimulate intrapulmonary EC activation and proliferation *via* the HIF-1/VEGF signaling pathway; (3) promote the Warburg effect through activation of the NF-κB/HIF-1α pathway (139); and (4) drive the high-mobility group protein 1 (HMGB1)/RAGE axis to promote EC-SMC vascular interactions in HPH (70). In addition, HIMF has angiogenic and vasoconstrictive properties that augment PAP and vascular resistance (140). This indicates that HIMF play a role in lung inflammation and vascular remodeling.

HMGB1 is frequently considered to be an atypical inflammatory cytokine. In experimental HPH, HMGB1 levels in serum (141) and pulmonary arteries were markedly elevated, which was associated with mPAP (68). Administration of exogenous recombinant HMGB1 worsened HPH, while treatment with HMGB1 inhibitors significantly attenuated the progression of HPH by recovering hemodynamic parameters, pulmonary vascular remodeling, and BMPR-II signaling pathways (69). Studies have demonstrated that HMGB1 promotes the proliferation and migration of PASMCs by inhibiting BMPR-II signaling and enhancing the expression of the pro-inflammatory factor IL-6 (142), thereby exacerbating the progression of HPH. Therefore, HMGB1 may be a potential therapeutic target for the prevention of vascular remodeling and pathology in HPH.

### 4.2 Chemokines

Dysregulated signaling of chemokines and their ligands has recently been linked to HPH development. In an animal model of chronic hypoxic PH, increased CCL2 production by pulmonary vascular cells contributes to increased chemotactic activity of monocytes/macrophages, which in turn activates outer membrane fibroblasts (143), stimulates PASMCs migration and proliferation, and induces pathological vascular remodeling. CCR2, a homologous receptor for CCL2, is also elevated in the lung tissue of HPH (25). CCR2 deficiency mediates pulmonary vascular wall lesions, and in the presence of hypoxia, results in more severe pathological changes owing to the activation of Notch signaling (144). However, Florentin et al (145) demonstrated that there was decreased pulmonary inflammation and reduced pulmonary vascular remodeling in CCR2-deficient mice, despite having no effect on hemodynamics. Despite these seemingly contradictory data, it is clear that the CCL2/CCR2 axis is a key signaling mechanism implicated in the pathological phase of PAH.

Increased CCL5 expression has been detected in several animal models of PH, including CH- (146), Su/Hx-, and HIMF-treated PH mice (147). CCL5 deletion significantly attenuated the development of HPH, as CCL5 deletion restored the function of BMP-mediated PAECs and reversed pulmonary artery angiogenesis and vascular occlusion. CCL5 has an upregulated receptor called CCR5, which is abundant in HPH in both vascular cells and lung tissues. Interestingly, the crosstalk between CCR5 and CCR2 mediates the collaboration between macrophages and PASMC, promoting inflammatory cell infiltration and PASMC migration and proliferation during PAH development (35).

The Y chromosome is protective against the development of hypoxia-induced PH in mice undergoing gonadectomy, as expression of the Y chromosome gene Uty suppresses CXCL9 and CXCL10, thereby attenuating the triggering of endothelial apoptosis and PH (148), whereas suppression of CXCL9 and CXCL10 reverses disease progression in multiple experimental models of HPH (149).

Total lung tissue expression of CXCL12 and its receptor, CXC chemokine receptor 4 (CXCR4), is elevated in animals subjected to chronic hypoxia (150). Progenitor cells, which modulate HPH and vascular remodeling, are mobilized and recruited to the pulmonary vascular system in both of these (151). CXCR7 is another receptor for CXCL12, and the CXCR7 antagonist CCX771 attenuates PH in newborn mice exposed to prolonged hypoxia by decreasing pulmonary vasculature remodeling and increasing oxygen delivery (152). However, treatment with CCX771 did not protect mice from developing chronic hypoxia-induced PH (150), suggesting that the pulmonary vascular microenvironment has a significant impact on CXCR7 function in HPH. In addition, the HIF-2α/CXCL12 axis is important for regulating pulmonary vascular homeostasis and pulmonary fibrosis. MK-6482 therapy, a Hif-2α inhibitor, attenuates HPH by reducing CXCL12 (153) production.

The inflammatory chemokine CX3CL1/fractalkine converts and activates CX3CR1+ leukocytes, such as monocytes/macrophages, γδT lymphocytes, and DCs, and the infiltration and accumulation of these immune cells in the lungs contributes to structural disruption and remodeling of the pulmonary vasculature. CX3CL1/Fractalkine expression was significantly upregulated in pulmonary microvascular endothelial cells from high-altitude individuals, patients with obstructive lung disease, and mice (33). CX3CL1 promotes the proliferation of pericytes and smooth muscle cells, which in turn aggravates pulmonary small-vessel myelination, causing pulmonary hemodynamic changes and RV hypertrophy (154). In addition, CX3CL1 can worsen HPH by regulating monocyte recruitment and macrophage polarization (25).

Briefly, cytokines, chemokines, and their receptors are involved in the progression of HPH and right ventricular remodeling. In addition, some cytokines are associated with disease severity and prognosis. Targeting these mediators may be a promising therapeutic strategy for HPH.

## 5 HIF linking inflammation and metabolism

Metabolic reprogramming (157) refers to the process by which tumor cells regulate their anabolic and catabolic metabolism to obtain the energy and substances required to survive in an extreme microenvironment. This process primarily involves the regulation of metabolic pathways involving sugars, lipids, and amino acids.

The Warburg effect, which represents metabolic reprogramming, is present in rapidly proliferating cells, such as many types of immune cells, and determines the role of immune cells in inflammation (158). Metabolic dysregulation of pulmonary vascular cells and immune cells is closely related to vascular lesions and structural changes in the right ventricle (159) Notably, HIF is a key reprogramming factor for inflammatory cell metabolism, and stabilization of HIF signaling contributes to the metabolic phenotypic shift in immune cells observed in PH.

Depending on their environment, immune cells with a strong plurality can polarize into a variety of functionally distinct phenotypes, with different polarization directions corresponding to different energy metabolism patterns. To react appropriately to various immunological responses, particularly in hypoxic inflammatory tissues, these cells adjust their specific energy demands under the regulation of HIF. Changes in energy supply patterns and cellular signaling pathways resulting from metabolic reprogramming promote pulmonary vasoconstriction and structural remodeling from multiple perspectives. Metabolism is an important node in HPH pathogenesis.

**Figure 2:**
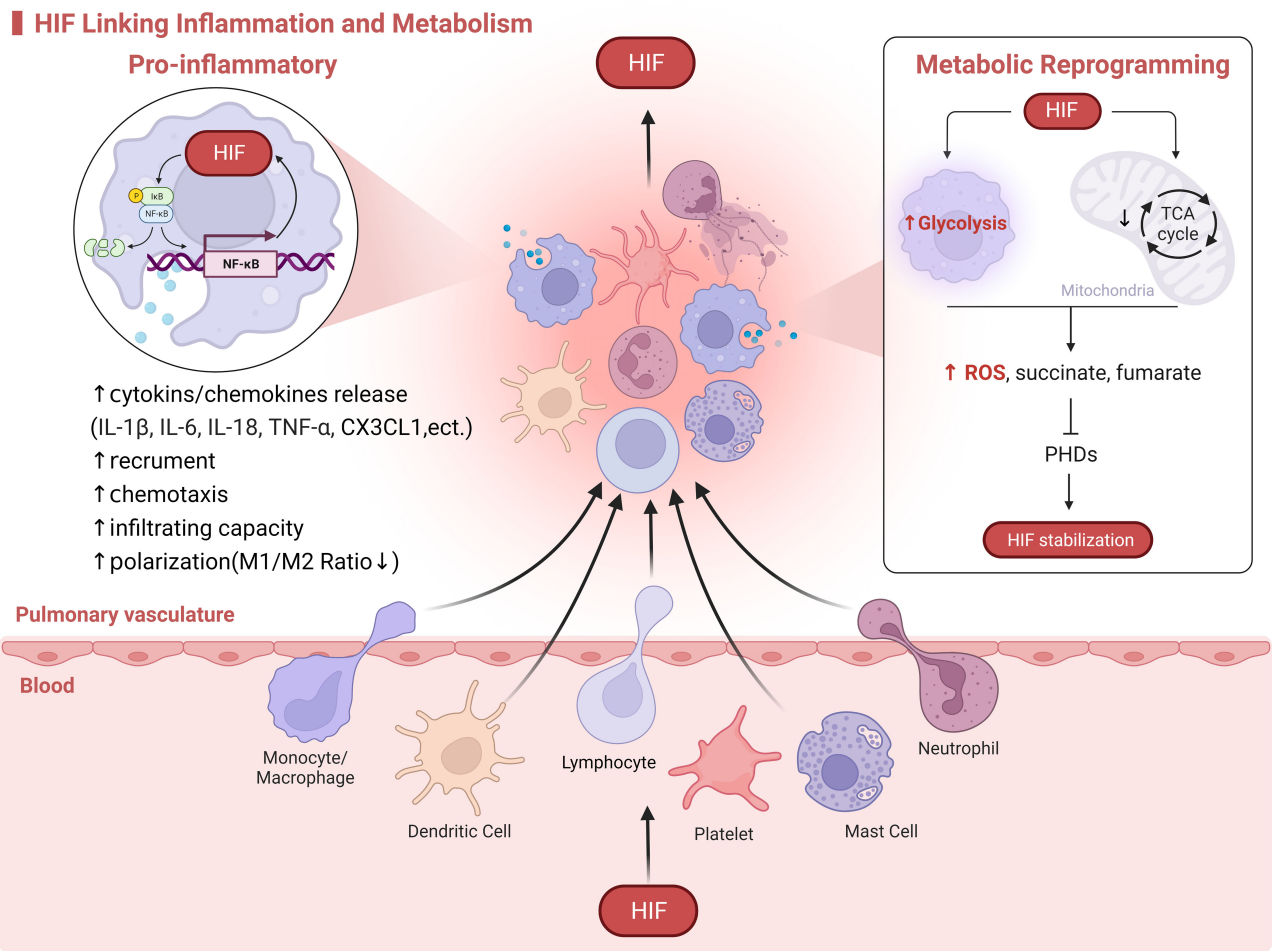
HIF Linking Inflammation and Metabolism. HIF serves as a molecular bridge in the immunoinflammatory and metabolism. On one hand, HIF promotes the activation of immune cells, metabolic reprogramming, and the release of inflammatory mediators. On the other hand, the exposure of immune cells to hypoxia from oxygen-deprived environments at sites of inflammation activates HIF. For example, in the case of macrophages infiltrating HPH, HIF drives the release of cytokines through crosstalk with the NF-κB signaling pathway. This enhances macrophage recruitment, chemotaxis, and polarization. Additionally, in response to hypoxia, mitochondrial oxidative phosphorylation is inhibited while glycolysis is enhanced. This leads to an increased production of ROS and metabolites such as succinate and fumarate by macrophages, which in turn activate and stabilize HIF.

Immunoinflammation and metabolic reprogramming are causally and mutually regulated, and HIF-1α may be a linking target of metabolic and inflammatory responses, with all three regulating the development of HPH (**Figure 2**).

## 6 Limitations of animal models

Hypoxia induces persistent vasoconstriction, pulmonary vascular remodeling, and development of PH during prolonged exposure. Hypoxic stress is a key driver of the pathogenesis of HPH. As a result, hypoxia is frequently utilized to induce PH in animal models and produce pathological features in cells (160). The small size, low cost, ease of maintenance, reproducibility, and consistency of rodent models mean that they are currently the primary source for understanding the molecular mechanisms of PH. The available HPH model construction methods include the chronic hypoxia model and the Su/Hx model.

Chronic hypoxia consistently induces PH in rodents (161), with both chronic sustained hypoxia (CSH) and chronic intermittent hypoxia (CIH) related to changes in PH and pulmonary vascular remodelling (162). Chronic hypoxia rarely causes muscular medial pulmonary artery hypertrophy and occlusive neointimal lesions and induces only mild-to-moderate PH (163). The PH and histological lesions are reversed when rodents return to normoxia. Therefore, the CSH model is well adapted to study less severe forms of PH, such as those associated with chronic lung disease and high altitude. Hence, it is a classical model for identifying cellular modifications that contribute to the pathogenesis of HPH. The degree of pulmonary vascular remodeling in response to hypoxia increases with the phylogenic order of the species (rat > mouse) (6). Compared to rats, pulmonary circulation in mice is relatively blunted in response to chronic persistent hypoxic stimuli, whereas PAP and pulmonary vascular remodeling induced by hypoxia are significantly lower (164). The microarray results suggest that this may be due to the fact that gene expression patterns are not identical between mouse and rat lung tissues (165).

CIH, which mimics the hypoxia-reoxygenation cycle characteristic of obstructive sleep apnea (166) has been found to cause PH in rodents (167, 168); however, the mechanisms underlying the differences between the effects of CIH in animal models and humans remain to be investigated.

The degree of hypoxia used in rodents is much more severe than that in humans, but a single hypoxic insult only causes minor vascular remodeling, which does not accurately mimic the severity of the disease in humans. Sugen 5416, an inhibitor of VEGFR2, combined with hypoxic stimulation, induces more severe PH pathological changes with an increased second hit, which contains inflammatory and vascular occlusive factors (169). Pathologically, SU5416 blunts VEGF receptors, leading to PAECs apoptosis (170). Disturbed by hypoxia, endothelial cell subpopulations and PASMCs proliferate further, and the combination of these actions leads to pathological vascular remodeling in the pulmonary circulation, elevated PA pressure, and right ventricular remodeling. Similar to PAH patients, Su/Hx rodent models have persistent and progressive PH (171), which differs from animals exposed to chronic hypoxia alone. Therefore, it may be more appropriate for the Su/Hx model to simulate a preclinical model of PAH rather than that of HPH.

In chronically hypoxic animals, several preclinical therapies were able to successfully prevent and reverse PH, but the majority of these treatments did not alleviate HPH. Therefore, the search for more robust animal models of HPH continues, and genetically engineered animals are promising tools for future pulmonary vascular studies.

## 7 Conclusion

In conclusion, the evidence points to the hypothesis that the pathophysiology of HPH is significantly influenced by immune response and inflammation. Histological analysis of HPH reveals how resident pulmonary vascular cells are affected by the extensive accumulation of immune cells and secretion of pro-inflammatory mediators. There is a causal connection between hypoxia and PH, and hypoxia interacts with the metabolic remodeling of immune cells and control of inflammation through the HIF signaling pathway. The establishment of a precise mechanism of action in pulmonary vascular pathology will open up a variety of novel targets for the intervention of HPH.

## Author contributions

YY conceived and drafted the manuscript. QX drew a graphic abstract. TW revised and supervised the manuscript and provided the financial support. All authors contributed to the article and approved the submitted version.
